# Correction: A mathematical model to quantify RYR Ca^2+^ leak and associated heat production in resting human skeletal muscle fibers

**DOI:** 10.1085/jgp.20211299404062022c

**Published:** 2022-04-14

**Authors:** Christopher J. Barclay, Bradley S. Launikonis

Vol. 154, No. 9 | 10.1085/jgp.202112994 | March 21, 2022

The initial version of Fig. A1 should not have included P3 and P7 data, and other data should have been merged. *JGP* regrets this error, which was introduced during the production process.

**Figure A1.**
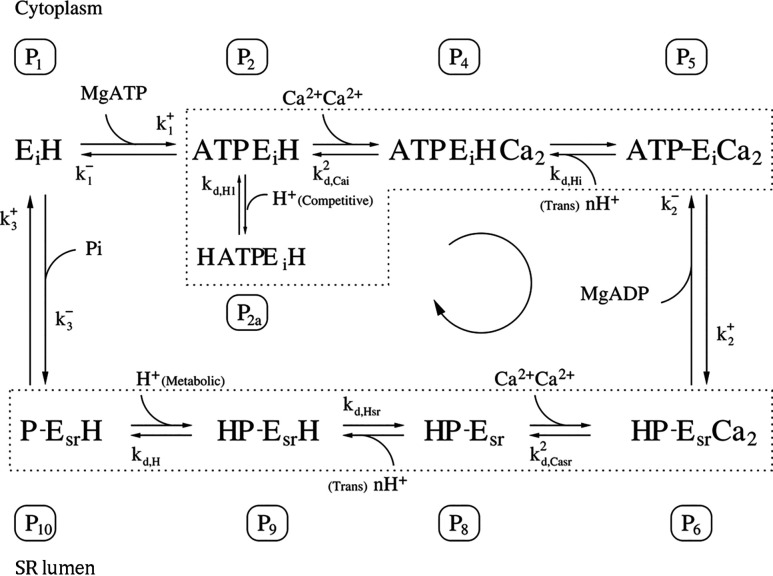


The corrected figure is shown here. The errors appear only in PDF versions downloaded on or before April 7, 2022.

